# Complete Genome Sequences of Lambdoid Phages 21, 434, and 434B and Several Lambda Hybrids

**DOI:** 10.1128/mra.00120-22

**Published:** 2022-04-12

**Authors:** Mike Feiss, Sankar Adhya, Costa Georgopoulos, Roger W. Hendrix, Graham F. Hatfull, Eddie B. Gilcrease, Sherwood R. Casjens, Jolene Ramsey, Ry Young

**Affiliations:** a Department of Microbiology, Roy J. and Lucille A. Carver College of Medicine, University of Iowa, Iowa City, Iowa, USA; b Laboratory of Molecular Biology, Center for Cancer Research, The National Cancer Institute, Bethesda, Maryland, USA; c Department of Biochemistry, University of Utah School of Medicine, Salt Lake City, Utah, USA; d Department of Biological Sciences, Pittsburgh Bacteriophage Institute, University of Pittsburgh, Pittsburgh, Pennsylvania, USA; e Department of Civil and Environmental Engineering, University of Utah, Salt Lake City, Utah, USA; f Division of Microbiology and Immunology, Pathology Department, University of Utah School of Medicine, Salt Lake City, Utah, USA; g School of Biological Sciences, University of Utah, Salt Lake City, Utah, USA; h Center for Phage Technology, Texas A&M University, College Station, Texas, USA; i Department of Biochemistry and Biophysics, Texas A&M University, College Station, Texas, USA; Queens College CUNY

## Abstract

Recombinational hybrids between phage λ and its relatives were instrumental in the beginnings of molecular biology. Here, we report the complete genome sequences of lambdoid phages 21 and 434 and three of their λ hybrids. In addition, we describe 434B, where the entire lysis gene region was replaced by cryptic prophage sequences.

## ANNOUNCEMENT

Escherichia coli phages 21 and 434, isolated by Wollman and Jacob in 1961 (1), were used to form genetic hybrids with the canonical phage λ. As they are close natural relatives, an analysis of these lambdoid phages and their hybrids was foundational to originating modern molecular genetics. Here, we report the complete genome sequences of phages 21 and 434; their λ recombinants λ *imm434*, λ *imm21*, and λ *h434 imm21*; and 434B, a clear plaque mutant of 434.

Phages were sourced as follows: phage 21, A. Campbell and R. Young; 434 wild type, S. Adhya; 434B clear mutant, C. Georgopoulos; λ *imm21* and λ *imm434*, M. Feiss (original source A. Campbell); and λ *h434 imm21*, C. Georgopoulos (original source of the *h434* host range allele, E. Signer). Phages were propagated on E. coli SKB-178 (2) by liquid infection in LB broth at 37°C. Virions were pelleted and purified by CsCl density step gradient centrifugation (3). Genomic DNA (gDNA) was isolated from purified virions with the phage DNA isolation kit (Norgen Biotek Corp., Thorold, Ontario) and sequenced individually by the Illumina MiSeq 150-bp paired-end run methodology with a 350-bp insert library prepared from a TruSeq DNA Nano kit at the University of Utah Sequencing Facility. Quality-controlled trimmed reads assembled into single >20-fold coverage contigs with Geneious 9.0.5 at default parameters and circular assemblies were reopened at known or homologous sticky ends (4–6). The phage 21 genome gave an identical assembly when sequenced by the dideoxy-nucleotide methodology at the Pittsburgh Bacteriophage Institute (7).

The genomes were annotated using the Center for Phage Technology Galaxy-Apollo phage annotation platform (8) with default parameters as follows: structural annotation, GLIMMER v3.0 and MetaGeneAnnotator v1.0 (9, 10); tRNA detection, ARAGORN v2.36 and tRNAscan-SE v2.0 (11, 12); gene function prediction, InterProScan v5.48, BLAST v2.9.0, TMHMM v2.0, LipoP v1.0, SignalP v5.0, and GenBank and Swiss-Prot databases, as well as HHPred using their HHSuite v3.0 Web server (13–20).

The phage 21 genome is 42,931 bp long with 73 protein-encoding genes and 2 tRNA genes. The 434 genome has 47,075 bp and 77 protein-encoding genes. The 434B clear plaque isolate was found to have a missense mutation in the *cI* repressor gene and a replacement of ∼5.5 kb, including the *Q* late activator, late promoter, and lysis gene region by a syntenic segment of the E. coli K-12 DLP12 cryptic prophage (21, 22). These sequences correct numerous single-base errors in previously reported segment sequences (Table 1). Essential genes in 21 and 434 are syntenic with phage λ, but the morons, including multigene loci encoding bacterial virulence factors, are different.

**TABLE 1:** List of data for the phages in this study

Phage in BioProject PRJNA222858	Genome length (bp)	GC content (%)	GenBank accession no.	GenBank accession no. of sequence fragments deposited previously	BioSample accession no.	Sequence Read Archive accession no.	Total no. of reads
21	42,931	51	OL657228	M81255, M23775, AJ237660, DQ372054, M58702, M65239, AH001308, AH007390, M61865, AF017628, EU078592	SAMN20971088	SRR15608961	662,329
434	47,993	50	OL657226	M12904, Y00118, M12803, X73093, J02460, V00635, M60848	SAMN20971089	SRR15608960	698,456
434B	47,075	50	OL657227		SAMN20971090	SRR15608959	229,839
λ *imm21*	46,148	50	OM418625		SAMN20971091	SRR15608958	241,494
λ *imm434*	47,326	50	OM418626		SAMN20971092	SRR15608957	299,503
λ *h434 imm21*	43,452	51	OM418627		SAMN20971093	SRR15608956	341,536

The sequences of the hybrid phages λ *imm21*, λ *imm434*, and λ *h434 imm21* confirm the genetically mapped locations of the nonhomologous 21 and 434 immunity segments, which confer host immunity to the same phage. The λ *imm21* and λ *imm434* hybrid sequences reveal additional 21 and 434 sequences, respectively, outside of the immunity regions; however, the interpretation of important early experiments using these phages is unaffected by these sequences (23, 24). The hybrid phage sequences identified 9 differences from the originally published λ sequence present in many extant laboratory λ strains (25).

### Data availability.

The genome sequences and associated data for the reported genomes are available in GenBank under accession no. OL657226 to OL657228 and OM418625 to OM418627, and sequence reads are available under BioProject PRJNA222858 (Table 1).

## References

[1] Jacob F, Wollman EL. 1961. Sexuality and the genetics of bacteria. Academic Press Inc., New York, NY.

[2] Georgopoulos CP, Hendrix RW, Casjens SR, Kaiser AD. 1973. Host participation in bacteriophage lambda head assembly. J Mol Biol 76:45–60. doi:10.1016/0022-2836(73)90080-6. 4578100

[3] Earnshaw W, Casjens S, Harrison SC. 1976. Assembly of the head of bacteriophage P22: X-ray diffraction from heads, proheads and related structures. J Mol Biol 104:387–410. doi:10.1016/0022-2836(76)90278-3. 781287

[4] Kearse M, Moir R, Wilson A, Stones-Havas S, Cheung M, Sturrock S, Buxton S, Cooper A, Markowitz S, Duran C, Thierer T, Ashton B, Meintjes P, Drummond A. 2012. Geneious Basic: an integrated and extendable desktop software platform for the organization and analysis of sequence data. Bioinformatics 28:1647–1649. doi:10.1093/bioinformatics/bts199. 22543367 PMC3371832

[5] Baldwin RL, Barrand P, Fritsch A, Goldthwait DA, Jacob F. 1966. Cohesive sites on the deoxyribonucleic acids from several temperate coliphages. J Mol Biol 17:343–357. doi:10.1016/S0022-2836(66)80146-8. 5336321

[6] Murray K, Murray NE. 1973. Terminal nucleotide sequences of DNA from temperate coliphages. Nat New Biol 243:134–139. doi:10.1038/newbio243134a0. 4515740

[7] Sanger F, Coulson AR, Barrell BG, Smith AJH, Roe BA. 1980. Cloning in single-stranded bacteriophage as an aid to rapid DNA sequencing. J Mol Biol 143:161–178. doi:10.1016/0022-2836(80)90196-5. 6260957

[8] Ramsey J, Rasche H, Maughmer C, Criscione A, Mijalis E, Liu M, Hu JC, Young R, Gill JJ. 2020. Galaxy and Apollo as a biologist-friendly interface for high-quality cooperative phage genome annotation. PLoS Comput Biol 16:e1008214. doi:10.1371/journal.pcbi.1008214. 33137082 PMC7660901

[9] Delcher AL, Harmon D, Kasif S, White O, Salzberg SL. 1999. Improved microbial gene identification with GLIMMER. Nucleic Acids Res 27:4636–4641. doi:10.1093/nar/27.23.4636. 10556321 PMC148753

[10] Noguchi H, Taniguchi T, Itoh T. 2008. MetaGeneAnnotator: detecting species-specific patterns of ribosomal binding site for precise gene prediction in anonymous prokaryotic and phage genomes. DNA Res 15:387–396. doi:10.1093/dnares/dsn027. 18940874 PMC2608843

[11] Chan PP, Lin BY, Mak AJ, Lowe TM. 2021. tRNAscan-SE 2.0: improved detection and functional classification of transfer RNA genes. Nucleic Acids Res 49:9077–9096. doi:10.1093/nar/gkab688. 34417604 PMC8450103

[12] Laslett D, Canback B. 2004. ARAGORN, a program to detect tRNA genes and tmRNA genes in nucleotide sequences. Nucleic Acids Res 32:11–16. doi:10.1093/nar/gkh152. 14704338 PMC373265

[13] Blum M, Chang H-Y, Chuguransky S, Grego T, Kandasaamy S, Mitchell A, Nuka G, Paysan-Lafosse T, Qureshi M, Raj S, Richardson L, Salazar GA, Williams L, Bork P, Bridge A, Gough J, Haft DH, Letunic I, Marchler-Bauer A, Mi H, Natale DA, Necci M, Orengo CA, Pandurangan AP, Rivoire C, Sigrist CJA, Sillitoe I, Thanki N, Thomas PD, Tosatto SCE, Wu CH, Bateman A, Finn RD. 2020. The InterPro protein families and domains database: 20 years on. Nucleic Acids Res 49:D344–D354. doi:10.1093/nar/gkaa977. PMC7778928 33156333

[14] Camacho C, Coulouris G, Avagyan V, Ma N, Papadopoulos J, Bealer K, Madden TL. 2009. BLAST+: architecture and applications. BMC Bioinformatics 10:421. doi:10.1186/1471-2105-10-421. 20003500 PMC2803857

[15] Krogh A, Larsson B, von Heijne G, Sonnhammer EL. 2001. Predicting transmembrane protein topology with a hidden Markov model: application to complete genomes. J Mol Biol 305:567–580. doi:10.1006/jmbi.2000.4315. 11152613

[16] Zimmermann L, Stephens A, Nam S-Z, Rau D, Kübler J, Lozajic M, Gabler F, Söding J, Lupas AN, Alva V. 2018. A completely reimplemented MPI bioinformatics toolkit with a new HHpred server at its core. J Mol Biol 430:2237–2243. doi:10.1016/j.jmb.2017.12.007. 29258817

[17] Juncker AS, Willenbrock H, Heijne GV, Brunak S, Nielsen H, Krogh A. 2003. Prediction of lipoprotein signal peptides in Gram-negative bacteria. Protein Sci 12:1652–1662. doi:10.1110/ps.0303703. 12876315 PMC2323952

[18] Armenteros JJA, Tsirigos KD, Sønderby CK, Petersen TN, Winther O, Brunak S, von Heijne G, Nielsen H. 2019. SignalP 5.0 improves signal peptide predictions using deep neural networks. Nat Biotechnol 37:420–423. doi:10.1038/s41587-019-0036-z. 30778233

[19] Sayers EW, Cavanaugh M, Clark K, Ostell J, Pruitt KD, Karsch-Mizrachi I. 2020. GenBank. Nucleic Acids Res 48:D84–D86. doi:10.1093/nar/gkz956. 31665464 PMC7145611

[20] The UniProt Consortium. 2021. UniProt: the universal protein knowledgebase in 2021. Nucleic Acids Res 49:D480–D489. doi:10.1093/nar/gkaa1100. 33237286 PMC7778908

[21] Campbell A. 1994. Comparative molecular biology of lambdoid phages. Annu Rev Microbiol 48:193–222. doi:10.1146/annurev.mi.48.100194.001205. 7826005

[22] Lindsey DF, Mullin DA, Walker JR. 1989. Characterization of the cryptic lambdoid prophage DLP12 of Escherichia coli and overlap of the DLP12 integrase gene with the tRNA gene argU. J Bacteriol 171:6197–6205. doi:10.1128/jb.171.11.6197-6205.1989. 2553674 PMC210489

[23] Werts C, Michel V, Hofnung M, Charbit A. 1994. Adsorption of bacteriophage lambda on the LamB protein of *Escherichia coli* K-12: point mutations in gene *J* of lambda responsible for extended host range. J Bacteriol 176:941–947. doi:10.1128/jb.176.4.941-947.1994. 8106335 PMC205142

[24] Kaiser AD, Jacob F. 1957. Recombination between related temperate bacteriophages and the genetic control of immunity and prophage localization. Virology 4:509–521. doi:10.1016/0042-6822(57)90083-1. 13507311

[25] Sanger F, Coulson AR, Hong GF, Hill DF, Petersen GB. 1982. Nucleotide sequence of bacteriophage λ DNA. J Mol Biol 162:729–773. doi:10.1016/0022-2836(82)90546-0. 6221115

